# The crisis of abstaining from self-cutting: a phenomenological study of three women's divergent trajectories after cessation

**DOI:** 10.3389/fpsyg.2026.1791431

**Published:** 2026-08-04

**Authors:** Anita Moe, Erik Stänicke, Hanne Haavind

**Affiliations:** Department of Psychology, University of Oslo, Oslo, Norway

**Keywords:** interpretative phenomenological analyses (IPA), recovery, self-cutting, self-cutting cessation, self-harm

## Abstract

**Background:**

Self-cutting is often treated as a symptom to be eliminated, with behavioral cessation regarded as a sign of therapeutic success. However, little is known about how individuals experience the absence of self-cutting after it has stopped—especially when the behavior has served complex symbolic, regulatory, and relational functions.

**Objective:**

This study explores how women with long-term histories of severe self-cutting experience the cessation of the behavior. It examines why abstaining from or stopping may not be experienced as recovery, but instead as a crisis, rupture, or new forms of suffering.

**Methods:**

Using Interpretative Phenomenological Analysis (IPA), we conducted and analyzed in-depth interviews with 12 women aged 18–35. All had engaged in repetitive self-cutting for several years and most reported having stopped or being abstinent at the time of the interview. Three cases—Solveig, Angelica, and Rachel—were selected for idiographic analysis to represent divergent ways of making sense of cessation.

**Results:**

Each woman experienced the end of cutting differently. For Solveig, cessation led to psychic collapse and emotional disintegration; the scars had functioned as containers of identity and trauma. For Angelica, cutting had regulated emotional extremes, and its absence exposed her to panic and somatic dysregulation. Rachel described a shift from cutting as a mode of survival toward a position in which meaning could increasingly be held in language and relationship rather than on the skin, allowing her scars to function as symbols of survival rather than sites of enactment. Rather than indicating a uniform trajectory of recovery, cessation marked a divergent and meaning-saturated transition shaped by how cutting had functioned psychically for each of them.

**Conclusion:**

The study highlights the psychological functions that cutting can serve, and the vulnerabilities that may arise when it stops. Clinicians should be cautious to equate behavioral change with psychological integration. Sustainable transitions away from self-harm require relational containment, narrative support, and symbolic substitution. Cessation may mark the beginning of deeper therapeutic work, not the end.

## Introduction

Self-cutting has been described in clinical literature for over a century (Emerson, 1913) and continues to pose challenges for clinicians and researchers seeking to understand its meanings and trajectories (Favazza, 1998; Moran et al., 2024). Within contemporary mental health care, non-suicidal self-injury (NSSI), including self-cutting, is often treated as a symptom to be reduced through structured interventions. Evidence-based treatments such as dialectical behavior therapy (Linehan, 1993; Linehan et al., 2006), mentalization-based therapy (Bateman and Fonagy, 2004), transference focused psychotherapy (Yeomans et al., 2015) and emotion regulation group therapy (Gratz and Gunderson, 2006) have demonstrated efficacy in reducing self-cutting frequency (Rossouw and Fonagy, 2012; Saunders and Smith, 2016), and symptom remission is frequently used as an outcome measure in clinical trials.

However, there is increasing recognition that behavioral cessation does not necessarily equate to psychological resolution or recovery. Large-scale longitudinal studies (e.g., Gunderson et al., 2011; Zanarini et al., 2012, 2024) show that individuals may stop self-harming while continuing to struggle with emotional dysregulation, identity disturbance, and interpersonal difficulties. Despite a growing body of research on the onset and maintenance of self-cutting, far less is known about how people stop, or how they experience the absence of self-harm once it ends. Until recently, few studies had examined the subjective and symbolic complexities of cessation (Mummé et al., 2017; Stänicke et al., 2020). A recent meta-synthesis of qualitative studies found that people who self-harm conceptualize recovery not as the absence of self-injury, but as an ongoing, relational, and meaning-laden process often marked by ambivalence, residual distress, and identity work (Lewin et al., 2024). Quantitative data likewise suggest that difficulty with cessation correlates with lower well-being and greater psychological struggle (Turner et al., 2022).

The discrepancy between clinical expectations and lived experience has received some attention in more recent qualitative literature. Several studies show that stopping self-harm can provoke identity disturbance, affective dysregulation, and loss (Shaw, 2006; Brown and Kimball, 2012). Others describe cessation as ambivalent or even destabilizing rather than relieving (Shaw, 2006; Gelinas and Wright, 2013; Mummé et al., 2017). A number of studies further highlight relational and contextual conditions that support or complicate recovery, such as peer validation, symbolic reframing, or narrative integration of past harm (Long et al., 2016; Lewin et al., 2024). Relatedly, sociological research has shown how the meanings of self-injury may persist beyond the act itself, for instance through scars or lingering images that continue to shape identity and experience after cessation (Gunnarsson, 2022, 2025). Still, few examine in depth how the *absence* of cutting is lived or how individuals make sense of that absence when cutting has served as an expression for pain, memory, or psychic coherence.

The current study builds on and extends this emerging body of work by asking not primarily how people stop, but what is lost when they do. In an earlier qualitative interview study (Moe et al., 2025; Moe and Ribe, 2007), we found that for individuals with long-term histories of self-cutting, the act served as a form of psychosocial labor on the skin, a way to express, care for, and symbolically mark what could not yet be spoken. Furthermore, we had observed that most of the participants shared an experience and a belief of having stopped cutting themselves. However, they described the cessation not as relief, but as rupture. In the present study, we return to these interviews, to explore their diverse experiences of abstention further.

By focusing on the paradoxical experience of cessation as crisis, we aim to contribute to a more nuanced understanding of what it means to stop cutting. Using an idiographic, phenomenological approach, we explore how this absence is lived and how the end of cutting is experienced over time. In contrast to models that frame cessation as linear or curative, our analysis traces the heterogeneity of post-cessation experience, drawing attention to the symbolic, communicative, and existential work performed by the act and its physical sequela, and the psychic disarray that may follow when it disappears.

We use the terms cessation and abstinence interchangeably to reflect both the participants' own experience and belief of having stopped self-cutting, and the temporal status of no active cutting at the time of interview. While most of the participants in the wider sample described themselves as no longer identifying as someone who cuts, several also acknowledged occasional relapses. The terms do not imply permanent abstention, but denote a period of discontinuation as subjectively experienced and reported.

The study is informed by the first author's clinical and research interest in self-cutting, developed through earlier work and clinical experience in out-patient and acute psychiatric settings. This background shaped the focus of the study, while the analysis aimed to remain close to the participants' own accounts.

## Method

### Recruitment procedure and participants

The Norwegian Regional Committees for Medical and Health Research Ethics approved the study. Patients with first-hand experience of cutting their skin over several years were invited to participate in a qualitative research interview about self-cutting. Recruitment was conducted through public outpatient mental health clinics in Norway. Participants were recruited through therapists working in mental health services. Therapists informed patients they knew had experience with repeated self-cutting about the study, and individuals who were interested were invited to contact the researcher directly. Participation was entirely voluntary and independent of ongoing treatment. The use of therapists in recruitment was intended to ensure that participants were approached in a clinically responsible manner. At the same time, this procedure may introduce a potential risk of gatekeeping, as therapists may influence who is informed about the study. This is considered a limitation of the recruitment process.

Participants were not selected based on any formal diagnostic criteria, and no diagnostic screening was conducted as part of the study, as our aim was to explore the phenomenon of self-cutting primarily, without restricting the sample to diagnosis-specific framework. All participants had been in long-term contact with mental health services, often over several years and with multiple therapists. None of the participants had a permanent job; most relied on public welfare support. Two were students. Participants' ages ranged from 18 to 35. Although both women and men were eligible for participation, only women volunteered. The present study cannot determine whether this reflects differences in prevalence, help-seeking, referral patterns, willingness to participate in qualitative research, or other factors. However, although both women and men engage in self-harm, earlier studies have reported that cutting is more commonly used by women, whereas men more often employ other methods (Laye-Gindhu and Schonert-Reichl, 2005; Whitlock et al., 2006).

All participants had a history of extensive self-cutting, ranging from several years to more than a decade. Interestingly, all but one reported that they had ceased or abstained from cutting at the time of the interview, ranging from a few months to years. None had been recruited on the basis of abstinence or cessation. Rather, participants differed markedly in how they described the experience of no longer cutting, and these differences gradually became an important focus of the analysis. In response to participant interest and thematic depth, four participants were interviewed more than once. All interviews were conducted by the first author.

### The interview

The interview format followed Haavind's (2000) guidelines for qualitative inquiry and used a semi-structured, exploratory format. An initial contract was established to ensure participants understood the nature and purpose of the study, with emphasis on the interviewer's interest in learning how participants understood and experienced their self-cutting. The interviews began with a prompt to describe a specific episode of cutting, in time and place, and from beginning to end. From there, participants were encouraged to reflect on what the act meant to them, how it felt, and how it related to their life situation, both past and present.

Although the interviewer did not explicitly ask about cessation, the topic emerged spontaneously in all interviews. Participants often raised the issue toward the end of the interview, with ambivalence or distress. As the interviews progressed, particular attention was given to the embodied, symbolic, and temporal aspects of the self-cutting process (cutting, bleeding, wounding, scabbing, and scarring) (Moe, 2005; Moe and Ribe, 2007), and to how participants experienced having stopped this sequence.

The interviews lasted from 90 min to 3 h. They were conducted in Norwegian, audio-recorded, and transcribed verbatim by the first author. English translations of excerpts have been made with attention to preserving the structure and tone of the original statements.

### Data analysis

The analysis was guided by principles of Interpretative Phenomenological Analysis (IPA), which emphasizes detailed, idiographic exploration of lived experience through an interpretative, hermeneutic process (Smith, 2004; Smith and Osborn, 2008). During the initial analysis of all twelve interviews, conducted for our previous study on the lived experience of self-cutting (Moe et al., 2025), we became aware of an unexpected phenomenon. Although the interviews primarily explored participants' experiences of cutting, several participants spontaneously reflected on what it was like to no longer cut. Compared with their often detailed descriptions of cutting itself, these accounts were typically more tentative, fragmented, and emotionally difficult to articulate. At the same time, they suggested that stopping was not necessarily experienced as relief or recovery, but could itself become a source of uncertainty, psychological distress, and profound disequilibrium. This unexpected observation became the starting point for the present study. This observation became the starting point for the present analysis. Returning to all 12 interviews, we examined participants' descriptions of abstinence and cessation in relation to their accounts of what cutting had previously meant to them. Rather than asking how participants had stopped cutting, we explored how the experience of no longer cutting illuminated different psychological functions that cutting had served.

As the comparative analysis progressed, we identified marked variation in how participants talked about and experienced abstention. To explore these experiential differences in greater depth, we returned to the full dataset and selected three cases that both illustrated contrasting experiential configurations and offered particularly rich reflections on the psychological meaning of cessation. Solveig and Rachel represented contrasting experiential poles, while Angelica exemplified an intermediate pattern that recurred across several interviews. The purpose of selecting these cases was not to develop a comprehensive typology of cessation, but to undertake an in-depth idiographic exploration of three contrasting experiential configurations. Although each participant's account remained unique, these experiential patterns were recognizable across the wider material and were most fully articulated in the selected cases.

Following the selection of the three focal cases, each interview was revisited repeatedly through close idiographic reading. Rather than coding for predefined themes, we sought to understand each participant's psychological world on its own terms, paying particular attention to how cutting, its absence, and the self were experienced and narrated. Through repeated comparison across the three cases, similarities and differences became increasingly clear, allowing us to refine our understanding of the distinct experiential configurations they represented.

Psychoanalytic developmental theory was introduced only after the phenomenological patterns had been identified, as an interpretative framework for articulating different modes of symbolization and psychological functioning. Theoretical concepts were therefore used to deepen, rather than generate or categorize, the empirical findings.

### Findings: Three experiential positions following cessation

In the analysis that follows, we present the three idiographic cases, Solveig, Angelica, and Rachel, to illustrate distinct experiential positions following abstaining from self-cutting. The accounts differ in how the abstention was lived, how cutting had functioned, and whether its absence brought fragmentation, disorganization, or transformation. Rather than seeking thematic saturation, we aim to illuminate the psychological diversity in how individuals inhabit the space where cutting no longer occurs but continues to matter.

#### Solveig: “I don't understand myself anymore”

Solveig was in her early twenties and lived alone with her 4-year-old daughter. She had grown up with a drug-abusing mother, experienced severe neglect, and was sexually abused by her father during her early teens. She had been in several abusive relationships and had lived in foster care. At the time of the interview, she described herself as not actively self-harming and had not cut herself since her daughter had become old enough to notice her body. This was the reason she offered for abstaining from cutting herself. Her interview was marked by long pauses, vivid metaphors, and moments of intense affect. Throughout, Solveig returned to the theme of cutting as a necessity for survival—an act that anchored her emotionally and cognitively. Having ceased this behavior, she now described her life as fragmented and frightening.

From the beginning of the interview, Solveig framed cutting not only as an act of self-harm, but as a communicative gesture. She distinguished between the location of the cuts and the message they carried:

When I've cut myself here (on the upper arm) it was more a matter of pain… but when I cut myself here (by the wrist) it was more a call for help, because I couldn't talk about it. The cutting was a very conscious act because I was so aware that I couldn't talk about it, but had to show it in another way.

The language here is precise and deliberate: “a very conscious act.” Her distinction between bodily sites reflects a clear awareness of how cutting functioned symbolically. For Solveig, the cut was a visible form of expression—an externalization of something that could not yet be spoken aloud. Her use of “because I couldn't talk about it” is repeated and emphatic, emphasizing the communicative breakdown that the cut temporarily resolved.

Later in the interview, when asked if she knew what the “it” was that she couldn't talk about, Solveig revealed the memory of her father's abuse:

I knew what it was. My dad abused me… It took a long time before I realized what was going on, really, because he gave me sleeping pills. So I didn't really understand it, it was like a dream, you know. I thought I was crazy, because it was like a dream. That's what hurt me, really. It was after that that I realized it. I probably started cutting myself towards the end, right before I realized it.

Here, cutting functions not just as communication to others, but as a form of internal realization. Her skin became the site where incoherent, dissociated memories could take shape. The scars, in this sense, preceded her conscious understanding of abuse. The cuts provided a kind of temporal bridge: they affirmed that something real had happened, even before she realized what had happened to her.

As the interview moved toward her present life, the tone shifted. Although Solveig was no longer cutting, she described her emotional life as more difficult than before:

Cutting has always helped me, but now… I don't understand myself anymore. When I used to cut myself, I was worse in a way, more suicidal, but I understood things. Now it feels as if I was stronger then, I did what I needed to do, now I don't. Things are so complicated now when I try not to, and everything gathers up inside of me. I hurt myself so badly psychologically. Because now… I have no outlet for my feelings.

Her account reverses the normative logic of recovery. Cutting was part of a functional, though precarious, psychic system. She describes a paradox: when she was actively cutting, she was “worse” but “understood things”. Now, despite apparent behavioral improvement, she feels weaker, disoriented, and unable to process emotion. Her description: “everything gathers up inside of me”, suggests an overwhelming internal pressure without containment. “I have no outlet” is repeated more than once during the interview, pointing to the vacuum left by the absence of cutting.

Later, Solveig elaborates on this experience with striking clarity: “Everything disappears, everything! I forget what I'm about to say; everything just disappears. And then when I want to cry, I can't, it doesn't work. I don't get rid of anything.” The rhythm and structure of her language mirror her described experience. Repetition (“everything… everything… everything”), short, interrupted phrases, and circularity (“I can't… it doesn't work”) all evoke a loss of narrative and representational capacity. The skin had functioned as a site for discharge and communication; without it, her emotional experiences become trapped and inert.

Throughout the interview, she returns to the importance of her scars. They are not merely markers of pain but carriers of meaning and memory. At one point, she says:

I feel… most of all, that I have paid for everything. That I really have been in pain, and I have the proof of it. On the other hand, I feel shame. It's so strange, so stupid, so ugly she is, the one who cuts herself. That's what I think. But then I think: I HAVE THEM HERE. The proof of what has happened.

Her internal dialogue is sharply polarized. Her scars are both a source of self-hatred and the only reliable affirmation of her suffering. The capitalized “I have them here” illustrates the embodied need to hold onto that proof. If she lets go of the scars, she loses not just a coping mechanism, but the only record of who she is and what she has endured.

The stakes of cessation become even clearer as Solveig describes her altered relationship to time:

I can never see myself as a different person than the one I was before I found out. I can't move on from being 13–14 years old. That's how I see myself. That's what's so strange. I haven't moved on. I'm still there. Everything. EVERYTHING. Everything about me, how I am. Clothes, makeup… it's almost like, the clothes today—I can't… I buy them because they're the only ones available. It's like time stands still. Completely still. Since before I found out.

This moment in the interview was marked by an emotional charge. Her repeated use of “everything,” her temporal anchoring in the age of trauma, and her ambivalence toward even mundane choices (like clothes) suggest a frozen self-state. The cessation of cutting has not allowed time to resume, but rather made her stuck within it. Without the cuts, she seems to have lost the temporal or affective narrative that used to organize her experience. Her psychic system, once stabilized by self-cutting and its bodily sequela, was left uncontained.

Toward the end of the interview, Solveig expressed a yearning to make sense of this disarray: “I'm hoping for an answer. Hoping that I'll figure it out, some words that will… that will… that will open everything, so that I'll understand everything, everything will be understandable… and everything will be fine. But it doesn't…” The repetition of “that will…”, followed by silence, seems to mark both desire and impossibility. She longs for a set of words that could do what the cutting and scars once did. Simultaneously, she acknowledges that the answer may never come.

Solveig's experience of cessation is not simply one of behavioral change, but of existential rupture. The cutting had provided her with access to a sense of continuity, coherence, and reality. In its absence, she is left with emotional flooding, narrative disintegration, and a fragile grasp on selfhood. Her story reveals the profound psychic labor that cessation demands—and the risk of collapse when that labor is unsupported and has not been properly done.

#### Angelica: “I have to keep on running”

Angelica was in her mid-twenties, lived with her boyfriend, and worked as a receptionist. She presented with a lively, talkative, and self-aware demeanor throughout the interview. She openly shared her strategies for avoiding inner states and reflected on the various forms her self-destructive behaviors had taken over time: cutting, starvation, drug use, and compulsive activity. Unlike Solveig, who described her scars as narrative anchors, Angelica's relationship with self-cutting was more pragmatic, even strategic. It served as a tool to manage overwhelming affect or, more often, a sense of absence.

She repeatedly described her inner life as marked by extremes: too much emotion or none at all. When asked about what made her cut, Angelica said:

Sometimes I cut myself because I didn't feel anything. My entire body was nothing. I used to say that I had so many thoughts that they all collapsed into a vacuum. Nothing was left, I couldn't think at all, there were no thoughts to think, and I had to cut to feel something.

The contradiction embedded in this statement: “so many thoughts” that lead to “no thoughts” seems to capture her cognitive and affective disintegration. Cutting appears not primarily a symbolic act here, but a method of “rebooting” the system.

Angelica also emphasized that cutting, like other behaviors she had relied on, was part of a broader attempt to maintain distance from emotional experiences and memories: “I thought those [cutting, doing drugs, starving myself] were clever things to do [laughing], everything is like… to distance myself, sort of…” The irony and laughter add a layer of detachment, yet the repetition of “everything is to distance myself” points to a persistent relational and intrapsychic dynamic. She is not trying to understand or resolve inner conflict, but to get away from it—quickly and completely. She later described her fear of stillness:

This last one, the therapist I've been seeing for 18 months, she's really pushing me, wanting to investigate why I can't deal with silence. Because I can't. I get a panic attack if I'm in a place where it's dark and quiet. I do not dare to sit down and face what might come.

Here, the avoidance is not of a specific memory, but of a more general unknown. “What might come” is left undefined, suggesting a diffuse but powerful fear. The cut, then, functions not to express trauma, but to interrupt its emergence.

At the time of the interview, Angelica said she had not cut herself for several months, a decision she linked to her relationship: “I wanted to stop when things got more serious between us [her and her boyfriend]. I wanted to try. But I live my life even more in a hurry now, constantly…” What follows is a list of destabilizing incidents: fainting at work, a car accident, an impulsive desire to break up. These events are not framed as isolated or accidental, but as symptomatic of something returning: “I thought I'd done well when I stopped starving myself, not doing drugs, not vomiting, not cutting… then this happens. I just don't get it.” Angelica's use of “this” is vague. Her reflection “I just don't get it” seems to signal both confusion and a longing to understand, yet there is little sense that she feels equipped to find meaning in these experiences.

Toward the end of the interview, Angelica describes the reemergence of memories from childhood: “It came over me. I started crying uncontrollably when I remembered. And then I got scared that there's more I don't remember. My psychologist said it might be, and that it will come when I'm ready. But I didn't feel ready at all.” Here, the language shifts from regulation and control to intrusion. Memories “come over” her, she had cried and feared returns. Without cutting, she seems more exposed to what her previous behaviors had kept at bay. The paradox is clear: stopping self-cutting was intended as progress, but it opened the door to psychic material she feels unequipped to handle.

Despite this, Angelica does express a sense of relief at being heard: “Just the fact that someone hears me… that helps, I guess. Since I haven't talked about it my whole life… just not having to think about it alone.” This is one of the few moments where relationality is foregrounded. Her tone is tentative (“I guess”) but the act of telling, of being accompanied in her story, carries meaning. This may hint at a shift from total self-reliance to the beginnings of symbolic containment through relationship.

Unlike Solveig, who invested heavily in her scars as bearers of history and identity, Angelica expressed a desire to separate from that part of her life. She commented: “I was never heard. I have a problem talking about this with my psychologist. It's a bit cliché. ‘I had a bad childhood because my mom didn't listen to me.' It doesn't sound serious. But it was serious. Really serious for me.” She downplays her story (“cliché”) even as she asserts its gravity (“really serious”). The repetition of “serious” acts as a self-reassurance in the absence of external validation.

Angelica's case illustrates a distinct kind of crisis. Where Solveig experienced psychic disintegration tied to unresolved trauma and a loss of identity, Angelica faced a collapse of regulatory structure. Cutting functioned less as symbolic inscription and more as affective regulation and interruption, a method to suppress, postpone, or displace emotional overwhelm. When she stopped, the symptoms did not disappear; they resurfaced somatically, relationally, and cognitively. She fainted, panicked, remembered, and dissociated.

Angelica's story illustrates that for some, cessation of self-cutting is not the end of struggle, but the beginning of a new and disorienting phase. Without the old structures in place, what remains is not simply recovery, but the rawness of unprocessed emotions and painful memories.

#### Rachel: “They [the scars] say I've survived”

Rachel was the oldest of the three participants, close to 30 years old. She lived alone, had a university degree, and was living on social welfare support at the time of the interview. Her demeanor was serious and calm, and she spoke in a structured and considered way. She made it clear from the outset that she did not want to share specific details about her past but gave enough context to hint to a history of trauma. Her narrative stood out for its degree of coherence and reflectiveness, and for the way she had come to assign meaning to her self-cutting over time.

Rachel was the participant who most explicitly and precisely described each stage of the self-cutting process: the gesture, the bloodshed, the wound, the scab, and the scar. Each of these phases held significance. Unlike most of the other participants, she had developed a symbolic language around her cutting and could trace how the meaning of the act had shifted as her inner world became more accessible.

She described the early phase of her self-cutting as a way to interrupt a pervasive sense of leaking:

When I was walking down the road, for example, it felt as if I were a sandbag full of holes whose entire content just ran out. I was a shell with less and less content. The entire me ran out, my personality, everything inside of me ran out. Then all that remained, all I was, was a shell of skin.

The metaphor reflects an experience of psychic leakage and emptiness. Rachel' sense of self appears porous and formless. The cut, in this context, offered a kind of material confirmation of internal existence: “Cutting explicitly showed me that you have feelings. It shows me there's something inside of me. Because when it feels like I've lost everything, the blood showed me that there was something left. I hadn't lost it all.”

Unlike Solveig, who used cutting to remember and mark trauma, and unlike Angelica, who used it to interrupt chaos, Rachel used it to verify presence. The blood was a proof of life, in the sense of affirming that something still resided within the boundaries of her skin.

Over time, however, this function shifted. As Rachel developed a therapeutic relationship characterized by persistence and safety, her narrative took on a new tone. The earlier self-cutting, which had provided verification of affect and existence, began to give way to relational experiences that could contain and reflect her internal states. “Eventually, I cut myself because I was feeling too much. In therapy I gradually realized that I was SO angry… it took years before I could admit to that.”

Here, the cut seems to change its function: from representing absence to expressing excess. Her awareness of anger, and the ability to locate it, name it, and assign it to relational contexts, reflects a movement toward symbolization. Importantly, Rachel acknowledged that this awareness was not immediately liberating, but painful:

That was a MAJOR setback, I thought, then. Because that was when it REALLY started to hurt. When you have a feeling and know something about why it hurts, then it's so extremely painful, and you know and there's nothing you can do about it.

This quote encapsulates the cost of psychological growth, where Rachel had entered a position where she could suffer with awareness, rather than evacuating affect through injury.

She also reflected on the communicative function of cutting, especially its power to elicit recognition from others:

To me, hurting myself is like… or it has become a ‘now I need someone else' kind of thing. There is a need, and I haven't been willing to acknowledge that need. This need has been associated with so much shame. But when you arrive at the emergency room with a 10 cm long cut, they too realize that you need someone else. It is so extremely concrete.

This formulation is notable for its clarity and emotional precision. Rachel recognizes the intersubjective dimension of her self-harm: the act makes visible what she cannot say and evokes a response that feels otherwise unreachable.

Over time, her need for care became expressible in words. Rachel described how a therapeutic relationship made it possible to leave the razorblade behind: “After years I was able to make a deal with him [her therapist], that I should no longer ‘cut out' what we were supposed to talk about during sessions.”

However, even with this developmental movement, Rachel acknowledged moments of longing. She missed the existential clarity the cutting once gave her; the urgency with which others responded and the directness of the scars. Reflecting on her scars, she said:

They say something about a healing process. They say that I've been through some rough times, but I've made it. Some people say that suicide is a cowardly thing to do. I don't think so at all, but it takes courage to live. And I've actually made it through all this. That's what gives me the guts to shower with other people. ‘OK, you can see that I've scars all over, but I've actually made it, I've survived.'

Rachel's final reflections underscored the irreducibility of lived experience and the value of storytelling: “It's impossible to fully explain what I've experienced, but I think it will be possible to explain it enough. I believe in the story, and in retelling. You need to, again and again, tell it to process it.” Her narrative reveals a transformation in how she uses words, symbols, and relationships to understand herself. She has moved from needing to mark pain to being able to speak it. Her scars remain, but they have changed in meaning. The absence of cutting, for her, is still ambivalent, but it is livable. Cessation, in her case, is not a collapse, but a shift from bodily survival to symbolic elaboration.

## Discussion: Abstention and cessation as crisis

The findings of this study suggest that cessation of self-cutting—while often framed clinically as progress—may, in lived experience, mark the onset of psychic crisis. All 12 participants described periods of abstinence not as signs of closure or relief, but as critical turning points that exposed them, to varying degrees, to emotional flooding, temporal disorientation, and destabilization of identity. These divergent accounts indicate that the absence of cutting is not experienced uniformly but is shaped by the psychic functions the behavior and physical sequelae previously served. To illustrate this variation, we presented three idiographic cases that exemplify different ways of experiencing cessation. This challenges linear models of recovery and aligns with recent research suggesting that cessation may not alleviate distress but rather transform its form and expression (Shaw, 2006; Lewin et al., 2024). In the discussion that follows, we examine these variations through two overlapping lenses: first, the symbolic and regulatory functions that cutting held for each participant; and second, the developmental positions from which cessation was experienced and negotiated. We then consider what these patterns may imply for therapeutic support when cutting stops, but psychic distress persists.

### Cessation as symbolic collapse: What cutting held together

The women's accounts highlight the various symbolic, affective, and relational functions that self-cutting had served, functions that were abruptly disrupted when the cutting stopped. For Solveig, the scars preserved a personal history that had no other witness. “I have them here, the proof,” she said, describing her fear that without visible signs of what had happened, her suffering would be erased. For Angelica, cutting was a means of regulating extremes, too much emotion, too little feeling. Cutting offered structure and control, even if only temporarily. Rachel, on the other hand, described the blood and wound as evidence that something *was* inside her, something that could be felt, named, and eventually expressed.

These differences suggest not only variations in personality or trauma history, but in symbolic functioning. Drawing on Segal's (1957) distinction between symbolic equation and symbolic representation, Solveig's scars appear to operate as symbolic equations, where the wound is equated with the experience itself. The scar *is* the abuse and its proof. This structure leaves little room for transformation. To remove or lose the scar is to lose access to history and self.

In Angelica's case, the cut appears less symbolically charged, functioning instead as a way to evacuate affect and divert herself. It interrupts unbearable psychic states without representing them. Her language is filled with metaphors of escape and collapse: “I have to run,” “I live my life even more in a hurry now,” “everything is to distance myself.” These expressions point to a limited capacity to link emotion to meaning. Bion (1962) describes such states as attacks on linking, a defensive refusal of symbolization. Angelica's post-cessation distress can be understood as the return of unlinked affect in the absence of her established regulatory strategy.

By contrast, Rachel demonstrates a shift in the symbolic value of the blood, wounds and scars. Initially, they verified her internal experience: “the blood showed me that I hadn't lost everything.” Later, through therapy, the scars became something that could be spoken, not just shown. She moved from body-language to verbal expression, and her scars were re-inscribed as signs of survival: “They say I've survived.” This shift reflects a growing capacity for symbolization and the integration of previously fragmented experience. Her trajectory suggests that meaning-making is possible when symbolic functions can be transferred from body to word, from private ritual to shared language.

The three participants also show different ways in which time and temporality are affected when cutting stops. For Solveig, time seems to freeze, her experience of herself stuck in a moment of realization, where stopping cutting has taken away a structure she used to hold herself together. Angelica, on the other hand, seems to speed up, almost as if she is trying to stay ahead of feelings or memories that might catch up with her if she slows down. Rachel's story is different again: She seems to move into a more ordinary sense of time, where her history can be remembered and put into words. These different relationships to time show how abstaining from self-cutting can affect how people experience time itself, not just emotionally, but structurally.

Some trauma theorists (e.g., Van der Kolk and McFarlane, 1996; Felman and Laub, 1992) have described how trauma can disturb the sense of time, and the participants in this study seem to show different versions of this. In that sense, cutting and the accompanying marks might have helped to locate experiences in time, as well as to regulate time, either by stopping it, speeding it up, or creating some kind of rhythm, and when that stops, people may be left with a kind of emptiness or confusion that needs to be worked through in new ways. This also adds depth to the findings from long-term outcome studies (e.g., Zanarini et al., 2024), which show that while many individuals report symptom reduction over time, they may still struggle with more existential aspects of recovery that often remain unaddressed.

Across these cases, the function of self-cutting spans a spectrum, from traumatic preservation to sensory regulation to symbolic transformation. When cutting ceases, what is lost is not merely a behavior, but an entire psychic infrastructure: a means of organizing time, affect, of constructing meaning, and of negotiating relational needs.

### Developmental positions and divergent trajectories

The women's divergent experiences of cessation appear closely tied to their underlying developmental positions, that is, to the ways in which they related to themselves, others, to their internal world and the marks of the self-cutting process. Each participant's experience of cessation was not only a response to losing a behavior, but to losing a function that had been deeply embedded in their psychic development. This variation aligns with psychoanalytic developmental theories that describe psychic structure as shaped by early relational patterns and internal representations (Klein, 1946; Bion, 1962; Winnicott, 1965).

While Solveig's experience might initially appear to reflect Kleinian dynamics, particularly the paranoid-schizoid position, her descriptions suggest something more primordial and less structured. She does not speak of persecutory fears or splitting between good and bad objects, but of a self on the verge of dissolution. Her narrative centers on a psychic and bodily fragmentation that lacks symbolic mediation. She describes states in which “everything disappears”, not through repression or defense, but through a collapse of internal cohesion. In this context, Ogden's (1989) concept of the autistic-contiguous position offers a more developmentally fundamental framework.

Ogden characterizes this position as a pre-symbolic mode of experience, where the self is organized around surface, sensation, and the integrity of the skin. Emotional states are not yet differentiated or represented, and the fear is not of annihilation by an object, but of psychic disintegration through uncontained stimuli. For Solveig, the cut seems to have functioned not as a communication to the other, but as a sensory and material intervention at the boundary of self, helping her hold together a minimal sense of cohesion (“Cutting has always helped me, but now… I don't understand myself anymore”). When the cutting ceased, she experienced not relief, but a dissolution of form and internal orientation. From this perspective, cessation represents not symbolic mastery, but a collapse of the one mechanism that gave some shape to an otherwise disorganized psychic field.

This function of cutting as a form of sensory boundary-making has been observed in several qualitative studies. For example, Brown and Kimball (2012) describe how individuals used the act of cutting to “stay inside themselves,” while Shaw (2006) identified the skin as a site of “inscription and containment.” Solveig's experience aligns with these perspectives, illustrating how the cut can serve as a last line of psychic demarcation.

Angelica's experience was less about psychic collapse and more about disconnection. She described a life of speed, distraction, and evasion. Her cutting was not loaded with symbolic meaning but served as a buffer against affective overwhelm and emptiness. When she stopped, the underlying disorganization resurfaced in somatic symptoms and panic. Bion's (1962) concept of attacks on linking is useful here: Angelica did not use cutting to represent or reflect, but to disrupt emotional processing altogether. Her crisis after cessation did not stem from the loss of memory or identity, but from the return of unprocessed affect without a container.

The centrality of emotional regulation in self-harm is echoed in several studies, and is arguably the most widely supported function identified in the literature (Klonsky, 2007). Gelinas and Wright (2013) found that many individuals continued to experience intense dysregulation even after cessation and often struggled to replace cutting with alternative strategies. Lewin et al. (2024) similarly report that the urge to cut may persist long after the act itself has stopped, especially when emotional overstimulation remains unprocessed.

Rachel, by contrast, appeared to be operating from what Klein called the depressive position: a developmental mode in which ambivalence, integration, and reparative capacity begin to emerge. Her narrative reflected self-reflection, symbolic elaboration, and a capacity to mourn the loss of cutting without collapsing. She acknowledged the longing but also engaged in a process of re-signifying her scars and experiences. This aligns with Bion's (1962) view that emotional suffering can only be transformed when it is symbolized and contained, rather than expelled or enacted. Thus, Rachel's story exemplifies how cessation can become part of a developmental transition, not just a behavioral shift. In the meta-synthesis by Lewin et al. (2024), participants who were able to “tell a story” about their self-harming history described greater psychological continuity and self-understanding.

These different positions reflect not only intrapsychic organization, but also the perceived availability and internal accessibility of relational support. Rachel emphasized the role of her therapist in co-creating language for her experience. Angelica, by contrast, repeatedly stated that she had “never been heard,” even though she acknowledged that her therapist encouraged her to share more of her experiences. Solveig described a history of being silenced by her parents and conveyed a persistent sense that she could not fully bring her pain into the therapeutic space. In both cases, the difficulty lay not necessarily in the absence of therapeutic engagement, but in the participants' limited capacity to use or trust it. The women's ability to transition away from cutting appears deeply connected to whether alternative forms of containment, such as relational recognition or narrative meaning, were not only offered but could be received and integrated.

The three experiential positions identified in this study also resonate with Stänicke's (2021) more recent clinically grounded differentiation of self-harm as the punished self, the unknown self, and the harmed self. While developed within a different empirical focus—namely adolescents' pathways into and out of self-harm—her framework offers a useful lens for situating the present findings. Our analysis does not, however, seek to classify participants according to ideal types, but to explore how previously established psychic configurations are exposed, destabilized, or transformed when self-cutting has ceased. In this light, Solveig's experience resonates with what Stänicke (2021) describes as the harmed self, Angelica's with aspects of the unknown self, and Rachel's trajectory with the punished self. At the same time, the present analysis extends this framework by showing how these positions become particularly visible—and clinically consequential—in the *absence* of self-harming. Cessation thus emerges not as a neutral endpoint, but as a developmental and phenomenological stress test of the psychic functions that self-cutting previously sustained. The present study highlights how cessation can mark very different psychic trajectories, depending on the symbolic, relational, and regulatory roles that cutting once fulfilled.

Finally, these trajectories should not be read as fixed or categorical. They represent psychic locations that individuals may move between over time (Segal, 1957; Britton, 2003). What this study highlights is that the experience of cessation is shaped not just by motivation or will, but by the psychic functions that cutting served—and by the patient's capacity to replace them with new, more symbolic forms of meaning-making.

### Therapeutic implications: What happens when the cutting ends?

The findings of this study carry several implications for psychotherapy with patients who self-injure, particularly those whose cutting is prolonged, repetitive, and severe. Most importantly, the study challenges the assumption that behavioral cessation of self-cutting automatically signals therapeutic progress. As seen across all three cases, stopping can trigger distress, psychic destabilization, and new forms of suffering.

For clinicians, this means attending carefully to what cutting has functioned as and how its loss might reverberate. A treatment approach that focuses narrowly on symptom elimination may overlook the profound existential role that cutting can play in a person's life. Solveig's case makes clear that for some, self-injury is not simply a behavior to remove, but a psychic scaffold: a means of preserving self-continuity in the aftermath of trauma. Therapeutic work in such cases must focus less on stopping the behavior and more on what replaces it. If there is no available symbolic or relational function to take its place, cessation may feel like annihilation.

Angelica demonstrates the necessity of recognizing cutting as part of a broader regulatory economy that may also include dissociation, somatic symptoms, relational distancing, or hyperactivity. Her case highlights the danger of assuming that the disappearance of cutting means stability has returned. For Angelica, stopping was followed by panic, fainting, and loss of affective control. These symptoms were not new, they were unmasked. Clinicians should be prepared for such reconfigurations and resist framing them as regression or resistance. Instead, they can be approached as a form of psychic exposure that requires new holding environments, whether relational, narrative, or somatic. This resonates with Turp's (2002) observation that self-harm may take less visible forms, including omissions in self-care that continue the psychic work once performed through more explicit and visible forms of self-harm.

Rachel's case offers an important counterpoint, showing that cessation can become an integrated and meaningful part of therapeutic change, but only when the symbolic and relational dimensions of cutting are explicitly addressed. Her therapist's ability to recognize and work with the meaning of her scars enabled her to shift from scars to words. This kind of work takes time, containment, and a sustained commitment to understanding rather than managing the behavior. Similarly, Brady (2014) emphasizes that cutting may function as an embodied narrative that requires interpretive listening before it can be symbolically relinquished.

These findings align with previous calls for more meaning-centered and relationally sensitive approaches to self-cutting, including work from both psychodynamic and sociological perspectives (e.g., Turp, 2007; Adshead, 2010; Motz, 2010; Steggals et al., 2022, 2024; Doctors, 1999). They support clinical models that treat self-injury not just as a dysregulation of affect, but as an attempt at symbolic communication and psychic survival (e.g., Stänicke, 2021). Turp (2002) and Medina (2011), for example, emphasize the need to engage directly with the meaning of self-harm in psychotherapy, including its links to shame, identity, and bodily experience. Similarly, Motz (2010) argues that self-harm can represent not despair but hope, a last effort to express what cannot yet be spoken. In this context, wounds and scars may also invite recognition and carry meaning beyond the act itself (Gunnarsson, 2022).

In this study, the cessation of cutting brought different challenges depending on each woman's developmental position, affective capacity, and relational context. One-size-fits-all treatment models, particularly those that define success as behavioral abstinence, may therefore be inadequate. Clinicians are encouraged to explore with patients not only why they harm themselves, but what that harm does for them, and what they fear will happen when it stops. This approach aligns with recent recommendations from Moran et al. (2024), which emphasize the need for individualized, meaning-centered care in the treatment of self-injury.

## Conclusion

Cessation of severe and prolonged self-cutting does not constitute a simple endpoint but a critical turning point, often experienced as crisis rather than recovery. In this study, three women's post-cessation trajectories illustrated how the absence of cutting exposed distinct forms of vulnerability, depending on the psychic functions the behavior had previously served. For some, cessation dismantled a fragile psychic scaffolding that had held trauma, identity, and temporal continuity together; for others, it unmasked affective chaos and somatic dysregulation; and for a few, it opened a space for transformation through symbolization and relational containment.

These divergent trajectories underscore that self-cutting cannot be understood merely as a maladaptive symptom to be eliminated. Rather, it may function as a form of psychic work: regulating affect, preserving continuity, externalizing unbearable experience, or sustaining a minimal sense of self. When cutting ceases, the work does not disappear. Instead, it demands new forms of containment, whether relational, symbolic, or narrative. In this sense, cessation acts as a developmental and phenomenological stress test, revealing both the limits and the possibilities of the individual's psychic organization.

By adopting an idiographic and interpretative phenomenological approach, this study highlights how experiences of abstinence are shaped not by motivation or will alone, but by underlying developmental positions and capacities for symbolization. The selection of three cases was guided by this idiographic logic, aiming to illuminate qualitatively different post-cessation configurations rather than to exhaust variation within the sample. The analysis thus contributes experience-near knowledge of how cessation may be lived as collapse, disorganization, or transformation, depending on what cutting had made possible before it stopped.

Clinically, these findings call for caution in equating behavioral cessation with psychological integration. For some patients, the insistence on stopping self-harm may precipitate psychic rupture if alternative forms of containment are not yet available. Psychotherapeutic work with patients who self-harm severely and over time must therefore attend not only to the behavior itself, but to its symbolic, affective, and relational meanings. Approaches grounded in psychoanalytic thinking may be particularly well suited to this task, as they offer concepts and clinical sensibilities for understanding how bodily enactments can gradually be translated into words, relationships, and shared narratives.

Finally, the study points toward directions for future research that are grounded in the phenomenon itself. Longitudinal and process-oriented designs are needed to explore how experiences of cessation unfold over time, particularly how crises following abstinence may persist, transform, or be resolved. Further work should also examine the therapeutic processes through which patients may move from bodily modes of expression toward more symbolic forms, and how clinicians can support this transition without inadvertently inducing destabilization. Such research would deepen our understanding of cessation not as an outcome, but as a vulnerable phase that requires careful listening, sustained containment, and co-symbolization.

Recovery, from this perspective, cannot be defined by absence alone. It must be understood as an ongoing process in which wounds are translated into words, scars into symbols, and trauma into experiences that can be held, thought about, and shared.

## Data Availability

The datasets generated and analyzed during the current study are not publicly available due to the sensitive nature of the interview material and the need to protect participant confidentiality. The data will be securely retained and destroyed in accordance with applicable ethical approvals and institutional data management requirements.
